# Operative Versus Nonoperative Management of High-Grade Acromioclavicular Injuries: A Systematic Review and Meta-Analysis

**DOI:** 10.7759/cureus.76682

**Published:** 2024-12-31

**Authors:** Maamoun Adra, Aslam Mohamed Haroon, Hugh Milchem, Shreehari Suresh, Yasmine J Khair, Haya El Merkabaoui, Rania Mansour, Mohamed Kamal M Youssef, Hayato Nakanishi, Christian Than, Rami Estfan, Greg Packer

**Affiliations:** 1 General Medicine, Peterborough City Hospital, Peterborough, GBR; 2 General Surgery, St George's University of London, London, GBR; 3 Surgery, University of Nicosia, Nicosia, CYP; 4 General Surgery, John Radcliffe Hospital, Oxford, GBR; 5 Orthopedic Surgery, American University of Beirut Medical Center, Beirut, LBN; 6 General Surgery, Mayo Clinic, Phoenix, USA; 7 Faculty of Medicine, Ain Shams University, Cairo, EGY; 8 Surgery, St George's University of London, London, GBR; 9 Biomedical Sciences, The University of Queensland, Brisbane, AUS; 10 Orthopedics and Trauma, Southend University Hospital, Essex, GBR

**Keywords:** acromioclavicular joint injury, meta-analysis, nonoperative, operative, rockwood classification iii-v

## Abstract

This meta‐analysis investigated differences between operative and nonoperative management for functional, as well as radiological, outcomes in Rockwood III-V acromioclavicular (AC) injuries. A literature search of several databases was conducted including Elsevier, Cumulative Index of Nursing and Allied Health Literature (CINAHL), PubMed, Cochrane Database of Systematic Reviews, Cochrane Central Register of Controlled Trials, Scopus, and Web of Science from inception to May 28, 2024. Included studies reported patients older than 16 years with a diagnosis of AC joint (ACJ) injury of Rockwood grade III or higher. This review was registered in PROSPERO (ID: CRD42023431602). Thirteen studies met the eligibility criteria (n = 729) of patients receiving either operative or nonoperative treatment for acute AC injuries. At 24-48 months follow‐up, constant score outcomes favored the operative group compared to the nonoperative group (MD = 2.38, 95% CI: 0.14, 4.62; I^2^ = 66%). Radiological outcomes were in favor of the operative group such that the ACJ width was narrower (MD = -5.60, 95% CI: -6.67, -4.54; I^2 ^= 11%), and the incidence of ACJ dislocations/subluxation was less compared to the nonoperative group (OR = 0.01, 95% CI: 0.00, 0.07; I^2 ^= 0%). More patients in the nonoperative group had “good” subjective evaluation of results compared to the operative group (OR = 0.55, 95% CI: 0.33, 0.90; I^2^ = 80%). There were 54 (18.3%) complications in the operative group. On the other hand, there were 39 (15.9%) adverse events in the nonoperative group. Operative management of Rockwood III-V AC injuries appears to confer greater functional and radiological patient outcomes. Further long-term research is required to elucidate whether this remains longitudinally, with specific investigation for individual Rockwood grading.

## Introduction and background

The majority of acromioclavicular joint (ACJ) injuries occur in young men between the ages of 20 and 40 years and are particularly common among athletes, accounting for 40%-50% of shoulder injuries in contact sports such as rugby, ice hockey, and judo [1-3]. The etiology is predominantly caused by direct trauma, resulting in separation or "dislocation" of the articulating surfaces, or indirect trauma causing a humeral head shift to impact the acromion [1,4].

Rockwood classification [5] is the most widely used system to grade ACJ injuries with grading based on the severity of ligamentous injury and degree of displacement of the distal clavicle from the acromion. Furthermore, the Rockwood grade is an important factor in determining the appropriate course of treatment. There is a consensus that low-grade, "low-energy" injuries such as Rockwood I and II should be managed conservatively [6]. The most accepted strategy involves brief sling immobilization followed by a rehabilitation program of strengthening exercises for the dynamic stabilizers of the joint and rotator cuff muscles [6,7]. Meanwhile, many surgical procedures, both open and arthroscopic approaches, have been described in the literature largely falling into two categories, either joint fixation or ligament reconstruction. Ligament repair and reconstruction include the modified Weaver-Dunn procedure as well as more recently pioneered methods using allografts and autografts [8,9]. On the other hand, fixation involves the use of screws, Kirschner wires (K-wires), hook plates, suture anchors, and various other materials to achieve reduction and primary fixation of the joint [10,11]. Lately, arthroscopic techniques using the "TightRope" device have been widely used as a functional nonrigid approach to ACJ repair [12].

Despite a wide array of approaches, controversy persists regarding the optimal management of "high" grade III, IV, and V injuries [13,14]. Traditionally, injuries of grade V or higher are treated operatively, while grade III injuries continue to be controversial [2,15]. However, recent studies have reported no difference in functional outcomes between nonoperative and operative treatment in high-grade AC injuries [16-20]. The benefit of nonsurgical treatment is the absence of complications that are associated with surgery. On the other hand, since the distal clavicle is usually not reduced to an anatomic distance, conservative methods can leave some patients with a greater-than-expected cosmetic deformity, persistent pain, or instability of the shoulder girdle [21,22]. Conversely, surgery is effective in achieving ACJ reduction while being associated with higher complication rates [22]. As such, this meta-analysis aims to compare functional and radiological outcomes as well as complications and return to previous activities between nonoperative and operative management of high-grade AC injuries to guide future practice.

## Review

Methods

Data Sources and Search Strategies

This review followed the Preferred Reporting Items for Systematic Reviews and Meta-analyses (PRISMA) guidelines [23]. A search of various databases from each database’s inception to May 28, 2024, was conducted with no language restrictions encountered. The databases searched include PubMed, Elsevier, Cumulative Index of Nursing and Allied Health Literature (CINAHL), Cochrane Central Register of Controlled Trials, Cochrane Database of Systematic Reviews, Scopus, and Web of Science. The search strategy was designed and conducted by a medical reference librarian. Controlled vocabulary with keywords was utilized to search for studies describing conservative versus surgical management of Rockwood III-V ACJ dislocations. The actual strategy which lists the search terms used and how they are combined can be found in Supplementary Material 1. This review was registered prospectively with PROSPERO (CRD42023431602). This study followed the PRISMA 2020 checklist [23] as provided in Supplementary Material 2.

Inclusion Criteria and Quality Assessment

Included studies must have met all the following inclusion criteria: (1) patients 16 years of age and above with ACJ injuries Rockwood grade III, IV, and V; (2) study designs such as randomized control trials, cohort studies, and case series; (3) have undergone surgical or nonsurgical intervention; (4) have undergone a rehabilitation program following intervention; (5) report on the primary outcomes; (6) employ a minimum follow-up period of six months; and (7) when two studies were reported by the same institution and/or authors, either the one of higher quality or the most recent publication was included in the analysis. Case reports, abstracts, conference abstracts, and studies with overlapping patient data were excluded. Article screening and data extraction were carried out by four independent assessors (AMH, HM, SS, YJK). The quality of each study was independently evaluated by three authors (YJK, SS, AMH) using the Newcastle Ottawa Assessment Scale [24]. Any disagreements were settled by MA and discussed among co-authors. The methodology and quality assessment are shown in Supplementary Material 3. The included studies had two-arm studies composed of operatively managed ACJ dislocations and conservatively managed ACJ dislocations. All studies included were acute AC injuries only.

Statistical Analysis

Means of continuous variables and rates of binary variables were pooled using the generic inverse variance method of DerSimonian and Laird [25]. Proportions underwent logit transformation before meta-analysis. The heterogeneity of effect size estimates across the studies was quantified using the I^2^ index (p < 0.10 was considered significant) and the Q statistic [26]. An I^2^ value of 0%-25% indicates minimal heterogeneity, 26%-50% moderate heterogeneity, and 51%-100% substantial heterogeneity. The model used was the random-effects model [26]. In studies in which mean and SD were unavailable, the median was converted to mean using formulas from the Cochrane Handbook for Systematic Reviews of Interventions [27]. If SD was unavailable or unextractable, the reported mean was removed from the calculation. Authors were contacted three times to obtain any relevant additional information that was not included in published articles. Funnel plots were used to visually assess publication bias [28]. Data analysis was performed using RevMan software version 5.4 (Review Manager (RevMan), The Cochrane Collaboration, 2020, Copenhagen, Denmark).

Outcomes and Data Extraction

Shoulder function and pain were the main outcomes of interest. Both were assessed via the Constant score [29] such that a higher score indicates better shoulder function and less pain perception. Constant scores were extracted in the following timelines: up to six months (short term) and between 24 and 48 months (medium term) postmanagement. Secondary outcomes including ACJ width (mm), radiological findings (n of events), complications/adverse events (n of events), return to previous activities and sports (n of events), and subjective evaluation of results (n of events) were also extracted at final follow-up and defined according to the included studies themselves.

Results

Study Selection and Characteristics

**Figure 1:**
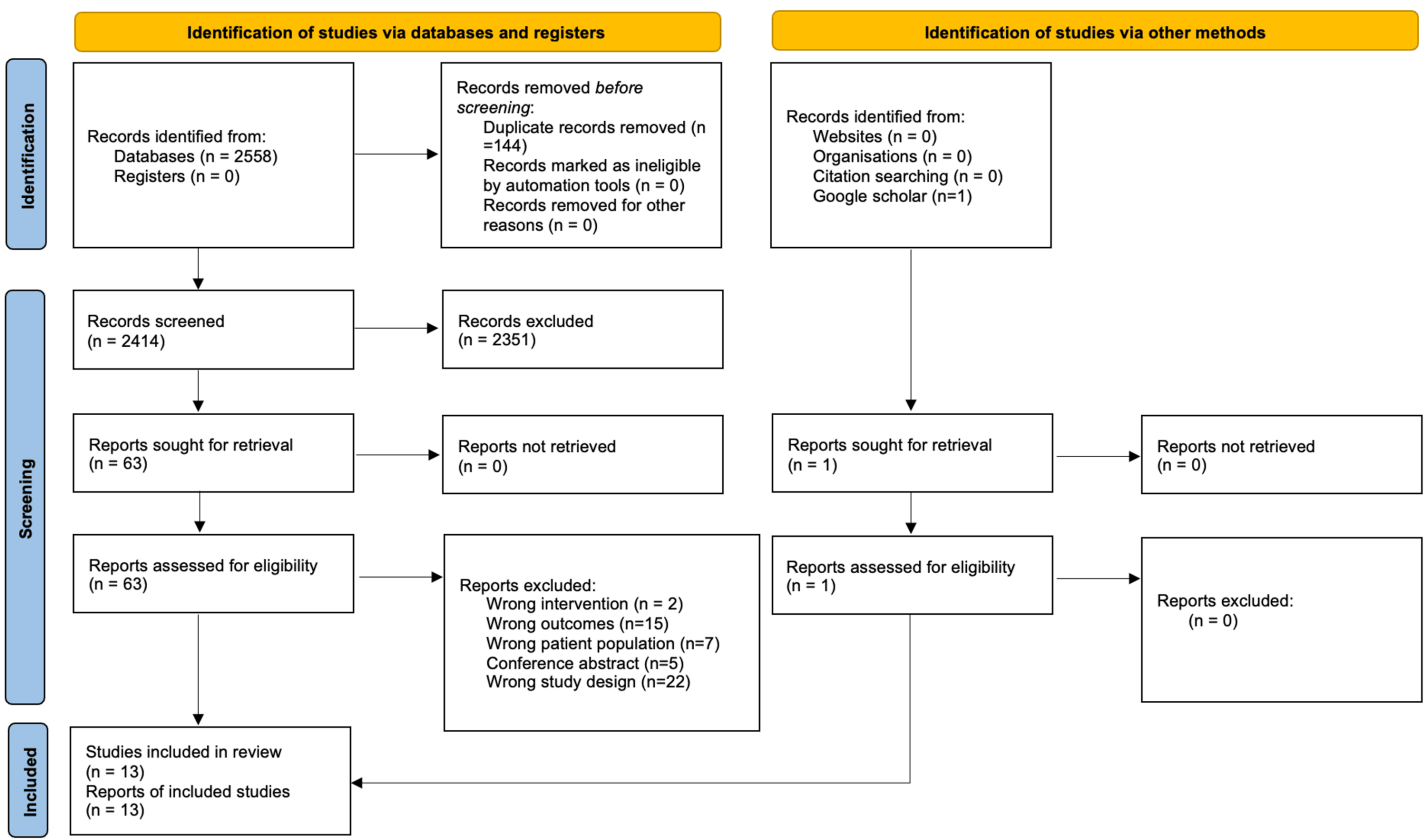
Preferred reporting items for systematic reviews and meta-analyses (PRISMA) flow diagram Source: [23]

The initial search yielded 2559 potentially relevant articles from which 13 unique studies involving 729 patients (males = 669, females = 60) met the eligibility criteria. The PRISMA flow chart (Figure 1) illustrates the details of the study selection process. The baseline characteristics of each included study are comprehensively described in Table 1. Three studies were cohort studies [30-32], seven studies were randomized control trials [17-19,33-36], one study was a retrospective case series [20], and two studies were reported as retrospective studies [37,38]. Five studies reported grade III injuries only [30,35-38], one study reported both grades III and IV [19], two studies reported both grades III and V [18,33], five studies reported grades III, IV, and V [17,20,31,32,34]. All included studies reported on acute AC injuries only [17-20,30-38]. The surgical techniques utilized varied across studies and included open reduction and internal fixation using TightRope [19,30,32,36,37], hook plate [17,18,34-36,38], K wires [33], coracoclavicular ligament (CLL) reconstruction via ligament augmentation and reconstruction system (LARS) [20], modified Weaver-Dunn technique [31], and suture button [20,31]. The surgical and nonsurgical treatment types and postoperation rehabilitation for each study can be found in Table 2.

**Table 1:** Baseline characteristics NR: not reported; SD: standard deviation; RCT: randomized control trial; ^†^this study is an updated study of COTS 2015 with the same patient population but new outcomes

Study	Country	Study design	Sample size			Male:female	Mean age ± SD (years)		Rockwood classification			Dominant side affected	Follow-up period ± SD (months)
			Total	Non-Op	Op		Non-op	Op	III	IV	V		
Álvarez-Álvarez et al., 2023 [30]	Spain	Retrospective cohort study	30	15	15	24:6	56.87± 16.86	44.27 ±15.02	30	NR	NR	15	36.83 ± 11.89
Boström Windhamre et al., 2022 [18]	Sweden	RCT	121	60	61	111:10	39.52 ± 10.81	39.51 ± 10.27	61	NR	60	91	Pre-set
Canadian Orthopaedic Trauma Society, 2015 [17]	Canada	RCT	83	43	40	78:5	37.3 ± NR	37.9 ± NR	70	3	8	50	Pre-set
Collaborative Orthopaedic Research Network, 2023 [31]	United Kingdom	Cohort study	54	38	16	49:5	38.2±15.4	37.7±13.4	29	7	18	27	Pre-set
De Carli et al., 2015 [37]	Italy	Retrospective study	55	25	30	55:0	28.5 ± NR	29.2 ± NR	55	NR	NR	NR	42 ± 18
Gstettner et al., 2008 [38]	Austria	Retrospective study	50	22	28	45:5	36.2 ± 12.6	37.2 ± 10.6	50	NR	NR	23	34.17 ± 12.26
Joukainen et al., 2014 [33]	Finland	RCT	25	9	16	23:2	54.0 ± 8.8	53.0 ± 7.8	11	NR	14	NR	226.13 ± 8.01
Mah et al., 2017^† ^[34]	Canada	RCT	-	-	-	-	-	-	-	-	-	-	-
Malik et al., 2021 [35]	Pakistan	RCT	94	47	47	89:5	30.1 ± 9.6	29.7 ± 9.6	94	NR	NR	NR	Pre-set
Murray et al., 2018 [19]	UK	RCT	60	31	29	56:4	30.0 ± 4.5	31.0 ± 4.0	36	24	NR	NR	Pre-set
Natera Cisneros et al., 2015 [32]	Spain	Retrospective cohort study	41	21	20	35:6	38.0 ± 9.0	36.00 ± 6.75	7	3	31	37	36.0 ± 16.5
Saade et al., 2023 [20]	France	Retrospective case series	38	14	24	37:1	42.8 ± NR	37.1 ± NR	19	6	13	19	29.0 ± 15.0
Tauber et al., 2023 [36]	Germany	RCT	78	39	39	67:11	38.4±12	39.3±13	78	NR	NR	37	Preset

**Table 2:** Treatment protocol and postoperative rehabilitation NR: not reported; CCL: coracoclavicular ligament; LARS: ligament augmentation and reconstruction system; NOS: not otherwise specified; K-wires: Kirschner wires

Study	Conservative treatment	Operative treatment	Postoperative rehab
Álvarez-Álvarez et al., 2023 [30]	Simple sling (without anti-rotation control device) for pain control, up to a maximum of three weeks. Isometric shoulder exercises were initiated with the aim of improving scapular stability. From the third week, passive and assisted exercises were started	TightRope system	Sling immobilization was preserved for three weeks along with scapular isometric exercises with increasing intensity initiated as tolerated. From the third week, passive and assisted mobilization exercises were started, allowing abduction and antepulsion above 90° after the 6 weeks postop, from which active mobilization began. The resisted activities were postponed until the twelfth week
Boström Windhamre et al., 2022 [18]	Collar "n" cuff for 10-14 days. Arm use was allowed for daily activities like eating, personal hygiene, as well as movement below the shoulder level with maximum loading of the arm of 1 kg, until 6 weeks after injury or surgery. After day 14, the home training program isometric muscle activation for the rotator cuff and controlled active movement training in a pain-free area (up to 90° flexion). After 6 weeks, free range of motion and loading of > 1 kilogram. Sports and heavy loading were allowed 3 months after injury or surgery	Hook plate	Same as a conservative treatment
Canadian Orthopaedic Trauma Society, 2015 [17]	Sling for 4 weeks followed by a physiotherapy regimen: active and passive exercise, followed by resisting and strengthening at 6 weeks after injury. Pendulum exercises were implemented at any time as dictated by the attending surgeon	Hook plate	Sling and formal physiotherapy were initiated 2 weeks postoperatively. Pendulum exercises were initiated based on the attending surgeon during this time. Active and passive exercises were initiated at this point, and the use of the sling was gradually discontinued. Resisted exercises were started at 6 weeks, and return to full activity was allowed at 3 months
Collaborative Orthopaedic Research Network, 2023 [31]	NR	Hook plating OR CCL suture button reconstruction OR modified Weaver-Dunn CCL suture reconstruction	NR
De Carli et al., 2015 [37]	Kenny Howard brace placement, ice therapy, and analgesia initially. Pendular exercises were permitted after 2 weeks. After 4 weeks, the brace was removed and shoulder motion was progressively increased as tolerated, initially using closed chain exercises and later using active exercises in open chain	TightRope system	Sling for 4 weeks. Pendular exercises were commenced within 2 weeks after the first visit. At 4 weeks, passive and active assisted range-of-motion (ROM), isometric, and closed chain exercises were begun. Active exercises through full ROM (ensuring scapula dynamic control) in an open chain (below the coronal plane) were started 8 weeks postoperatively. Contact sports and heavy work were permitted after 12 weeks
Fraschini et al., 2010 [39]	NR	Dacron vascular prosthesis OR LARS artificial ligament	Sling for 2 weeks and K wire removal after 3 weeks
Gstettner et al., 2008 [38]	Sling, analgesia, and physical therapy until a free range of movement was achieved (NOS)	Hook plate	Sling for 4 weeks, physical therapy, including passive motion twice a week (NOS). After 4 weeks, the patient was allowed to elevate and abduct the arm actively to a level of 90. The plate was removed after 12 weeks
Joukainen et al., 2014 [33]	Kenny-Howard splint for 4 weeks. Encouraged to mobilize the elbow several times per day, and mobilization of the shoulder with pendulum-type movements was initiated 4 weeks after the injury. Active mobilization of the shoulder was allowed 6 weeks after injury	Open reduction and fixation with 2 smooth Kirschner wires	Sling for 4 weeks and mobilization of the shoulder was allowed after 4 to 6 weeks in a similar manner as in the nonoperative group. The K-wires were removed at 6 weeks postoperatively
Mah et al., 2017 [34]	Sling and a standard physiotherapy regimen (NOS)	Hook plate	Sling and physiotherapy (NOS). Hook plate removal after 6 months at either the patient’s request or the attending surgeon’s discretion.
Malik et al., 2021 [35]	Collar and cuff sling for 3 weeks followed by physiotherapy consisting of graduated exercises (NOS)	Hook plate	Collar and cuff sling which was removed after 2 weeks and physiotherapy was started consisting of graduated exercises (NOS)
Murray et al., 2019 [19]	Sling for 3 weeks and passive circumduction exercises without the sling 4 times per day. After the sling was discarded, the physiotherapist-led rehabilitation program began with active shoulder motion exercises	TightRope system	Same as conservative treatment + unrestricted range of motion was allowed beginning at 6 weeks postoperatively. Patients were encouraged to perform strengthening exercises (NOS) until 1 year postoperatively. Patients were advised against competitive or contact sports until 3 months postoperatively
Natera Cisneros et al., 2015 [32]	Sling for 3-4 weeks, anti-inflammatories, and physical therapy regimen: initially allowed to move fully and actively the elbow, wrist, and hand. Allowed passive movement of the shoulder no more than 90° of elevation in the plane of the scapula after the first week. Pendulum exercises begin from the first week postinjury. The active range of motion was progressively advanced from the sixth week onward. Exercises to regain strength were initiated once the patient had a full, pain-free passive, and active range of motion, and exercises were primarily directed toward scapular stabilization	TightRope system	Sling for 3–4 weeks and physical therapy regimen: initially allowed to move fully and actively the elbow, wrist, and hand. Allowed passive movement of the shoulder no more than 90° of elevation in the plane of the scapula 3 weeks after surgery. Pendulum exercises begin in the third week postsurgery. The active range of motion was progressively advanced from the sixth week onwards. Exercises to regain strength were initiated once the patient had full, pain-free passive, and active range of motion, and exercises were primarily directed toward scapular stabilization
Saade et al., 2023 [20]	Sling to relieve pain (length of immobilization was not defined and left up to the patient) Physical therapy (NOS) was not always prescribed	Synthetic ligament or endo-button	Shoulder immobilizer with the elbow internally rotated at the side for 4 to 6 weeks. After this immobilization period, a rehabilitation specialist was tasked with helping the patient progress from passive to active-assisted to active range of motion, two to three times per week plus self-directed rehabilitation at home. Muscle strengthening (NOS) was started only in the 3rd month postoperatively
Tauber et al., 2023 [36]	Sling, adequate pain management using nonsteroidal antirheumatics for several days, and local ice therapy. Duration of immobilization was based on the patient’s pain level and lasted between 10 and 14 days. Physical rehabilitation measures were initiated under a physiotherapist’s guidance, performed for 6 to 8 sessions with a frequency of 2 times per week, and continued until full range of motion (ROM) was achieved. The protocol was allowed to be adjusted individually to the pain level of the patient (NOS)	TightTope system OR hook plate	Pain management, local ice therapy, and sling for 6 weeks. During this period, only passive motion and exercises until 90° of glenohumeral abduction were allowed. Actively assisted shoulder motion in all planes and muscle strengthening followed for the next 6 weeks, with shoulder sports starting after 4 months

Risk of Bias

Results of the quality assessment of all included studies are shown in Supplementary Material 3. The Newcastle-Ottawa quality assessment scale was used to evaluate the risk of bias and quality of included studies. Each study was categorized into good, fair, and poor quality according to the scores attained in the selection, comparability, and outcome/exposure domains. All 13 studies were judged to be of good quality. The patients appeared to represent the whole experience of the investigator, the exposure and outcome were adequately ascertained, and the lengths of follow-up were adequate to observe any change in the clinical outcomes.

Mechanism of Injury

There were a variety of mechanisms of injury documented. The most common mechanism of injury was sports injury, including soccer, handball, rugby, sprinting, and other sports (not otherwise specified), which was reported in six studies accounting for 164 out of 397 AC injuries in total [17-20,31,32]. Six studies reported 128 patients out of 384 who sustained an AC injury by falling at the same level [17-19,31-33]. Four studies reported 100 patients out of 283 who sustained the injury due to a motorcycling accident [17,18,20,32]. Five studies reported 88 patients out of 330 who were injured due to a cycling accident [17-19,32,33].

Constant Scores

**Figure 2:**
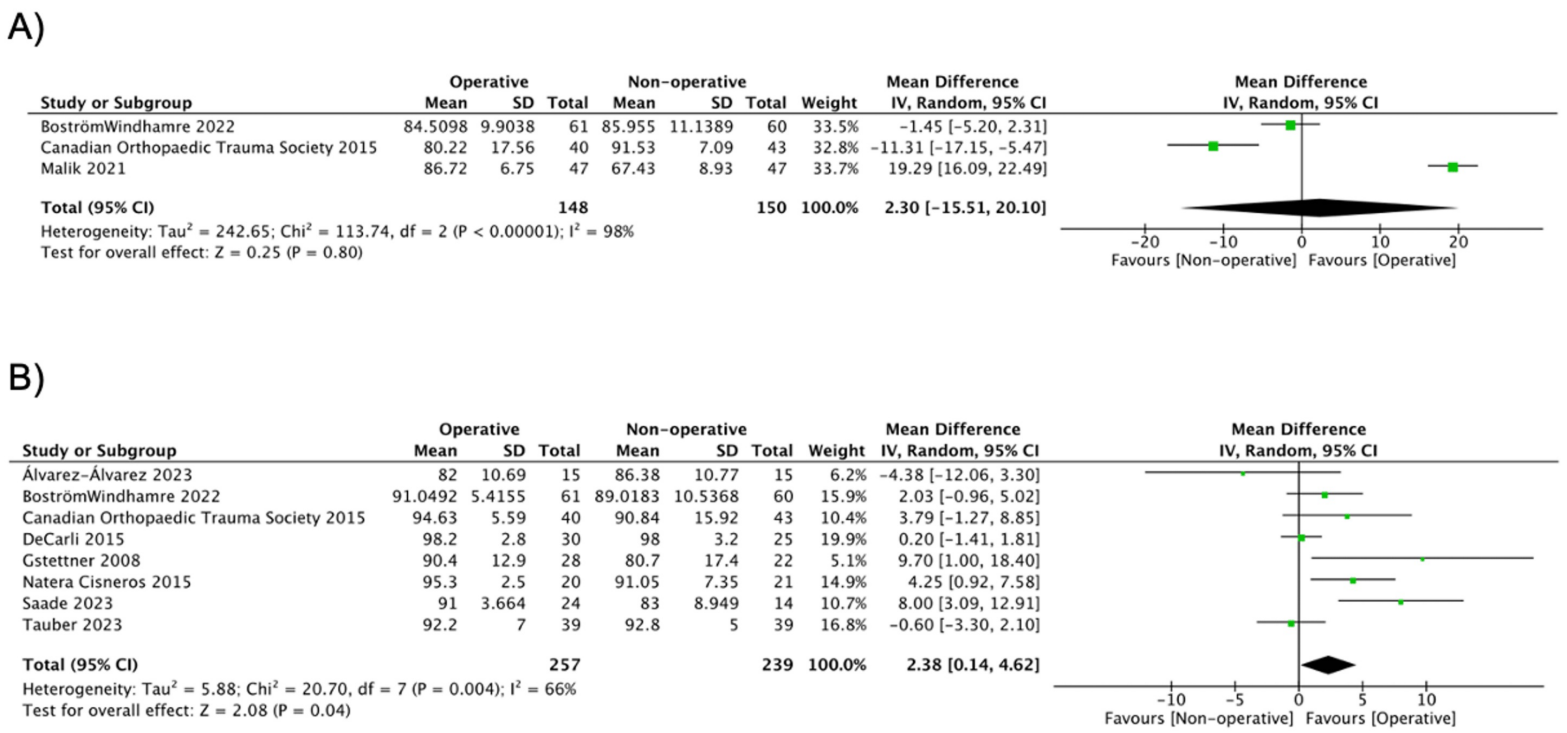
Pooled estimate of Constant score for operative vs. nonoperative group, (a) up to six months and (b) between 24 and 48 months (A) Source: [17,18,35] (B) Source: [17,18,20,30,32,36-38]

Constant scores at different time points are summarized in Table 3. At six months follow-up, no difference was observed between the two groups (MD = 2.30, 95% CI: -15.51, 20.10; I^2^ = 98%) [17,18,35]. Between 24 and 48 months of follow-up, the Constant scores were in favor of the operative group (MD = 2.38, 95% CI: 0.14, 4.62; I^2 ^= 66%) (Figure 2) [17,18,20,30,32,36-38].

**Table 3:** Reported continuous outcomes SD: standard deviation; N: study sample; n: sample size

Outcomes	Up to 6 months						24-48 months					
	Operative			Nonoperative			Operative			Nonoperative		
	Mean ± SD	N	n	Mean ± SD	N	n	Mean ± SD	N	n	Mean ± SD	N	n
Constant score	84.05 ± 11.94	3	148	81.75 ± 13.69	3	150	92.34 ± 7.62	8	257	89.80 ± 11.83	8	239
ACJ width (mm)	-			-			5.41 ± 3.70	3	73	10.67 ± 4.56	3	62

ACJ Width and Radiological Outcomes

**Figure 3:**
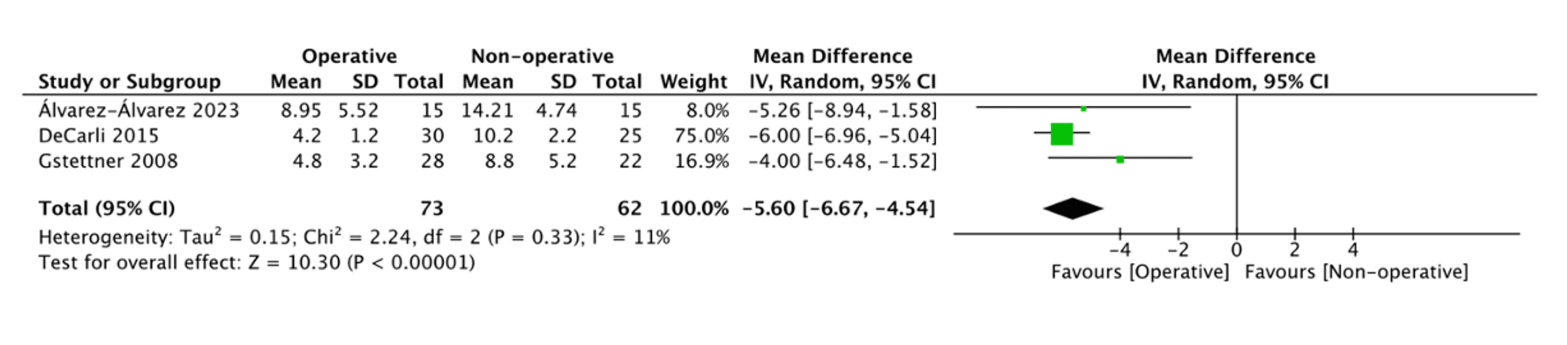
Pooled estimate of acromioclavicular joint (ACJ) width for operative vs. nonoperative group at up to 48 months Source: [30,37,38]

**Figure 4:**
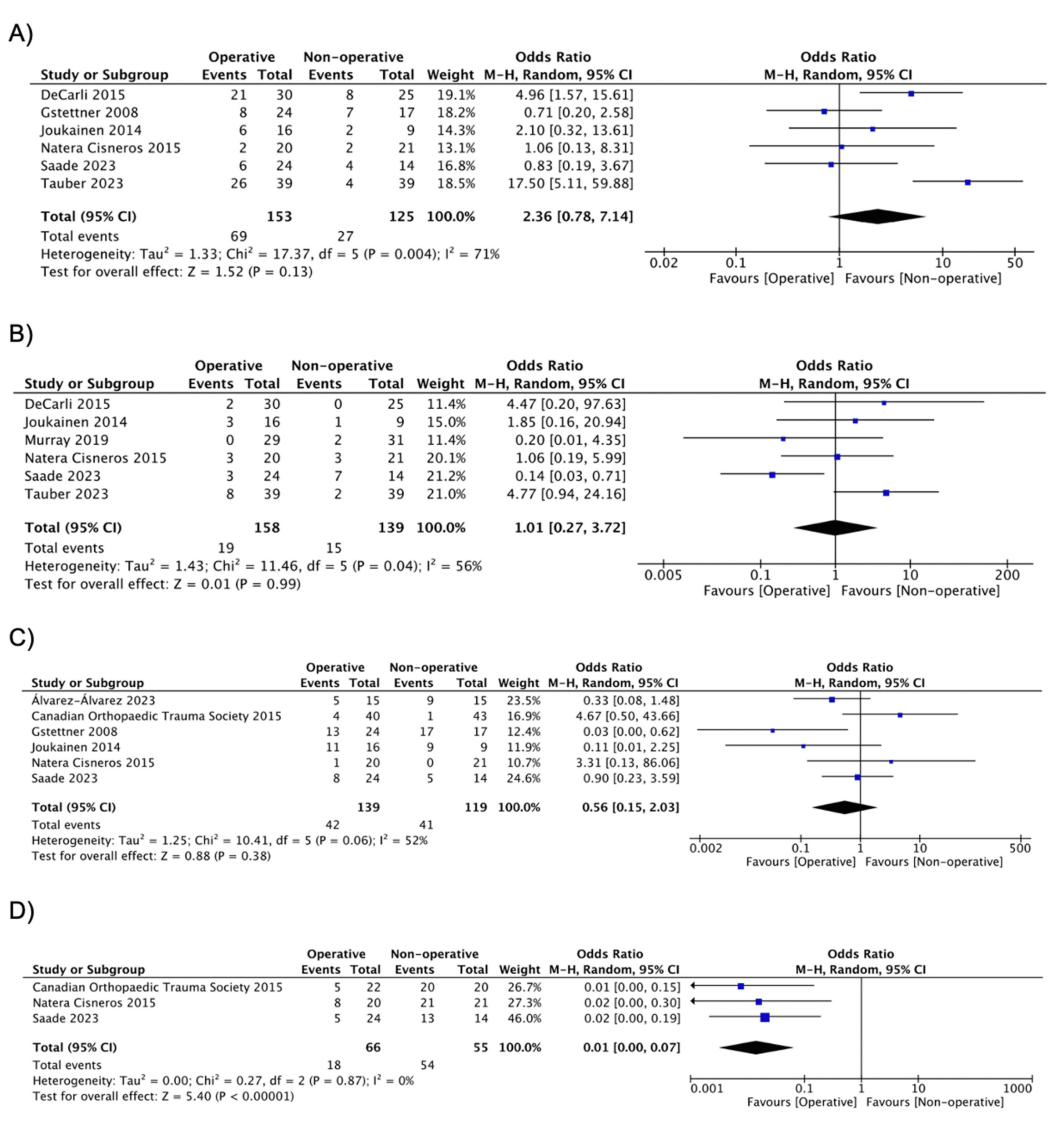
Pooled estimate of radiological outcomes for operative vs. nonoperative group: (a) calcifications/ossification of CLL, (b) osteolysis, (c) degenerative changes, (d) ACJ dislocation/subluxation CLL: coracoclavicular ligament; ACJ: acromioclavicular joint Source: (A) [20,32,33,36-38], (B) [19,20,32,33,36,37], (C) [17,20,30,32,33,38], (D) [17,20,32]

AC distance at 24 to 48 months follow-up is summarized in Table 3. There was a difference in favor of the operative group (MD = -5.60, 95% CI: -6.67, -4.54; I^2 ^= 11%) (Figure 3) [30,37,38]. Radiological outcomes are summarized in Table 4. No difference in the incidences of calcifications/ossification of the coracoclavicular ligament (CLL) (OR = 2.36, 95% CI: 0.78, 7.14; I^2 ^= 71%) [20,32,33,36-38], osteolysis (OR = 1.01, 95% CI: 0.27, 3.72; I^2^ = 56%) [19,20,32,33,36,37], and degenerative changes (OR = 0.56, 95% CI: 0.15, 2.03; I^2 ^= 52%) [17,20,30,32,33,38] was seen. The presence of ACJ dislocation/subluxation was in favor of the operative group demonstrating fewer incidences (OR = 0.01, 95% CI: 0.00, 0.07; I^2 ^= 0%) (Figure 4) [17,20,32].

**Table 4:** Radiological outcomes ACJ: acromioclavicular joint; CLL: coracoclavicular ligament; OR: odds ratio; CI: confidence interval; * statistical significance

Radiological outcomes	Events/sample size		-			
	Operative	Nonoperative	OR	95% CI	I^2^	Included study groups
Calcifications/ossifications of CLL	69/153	27/125	2.36	0.78-7.14	71%	6
Osteolysis	19/158	15/139	1.01	0.27-3.72	56%	6
Degenerative changes	42/139	41/119	0.56	0.15-2.03	52%	6
ACJ dislocation/subluxation	18/66	54/55	0.01*	0.00-0.07	0%	3

Return to Work and Sports

**Figure 5:**
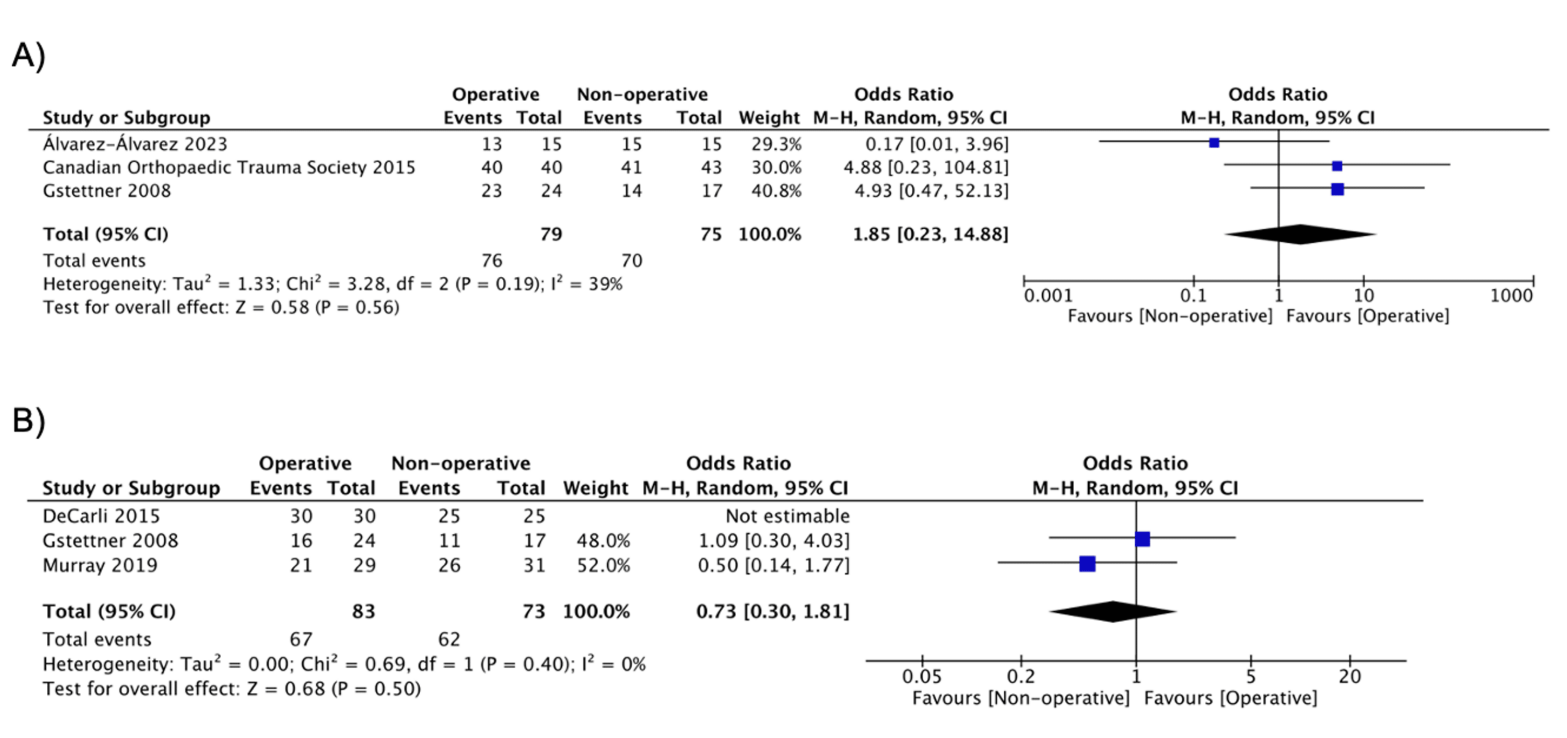
Pooled estimate of (a) return to work/previous activity and (b) return to sports for operative vs. nonoperative group Source: (A) [17,30,38], (B) [19,37,38]

Return to work and sports are summarized in Table 5. At the final follow-up, there was no significant difference in return to work (OR = 1.85, 95% CI: 0.23, 14.88; I^2^ = 39%) [17,30,38] or return to sports (OR = 0.73, 95% CI: 0.30, 1.81; I^2^ = 0%) (Figure 5) [19,37,38].

Subjective Evaluation

**Figure 6:**
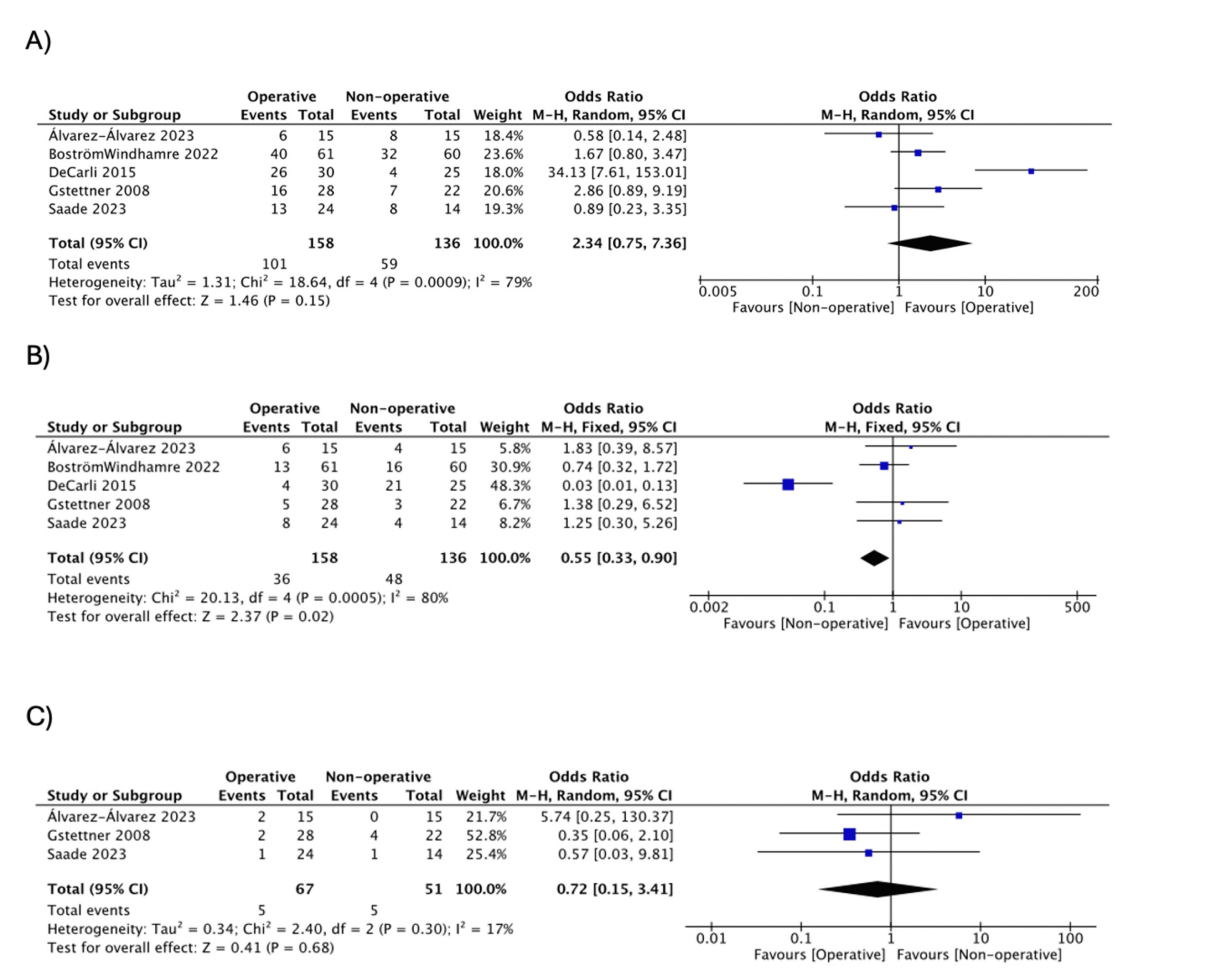
Pooled estimate of subjective evaluation for operative vs. nonoperative group: (a) excellent, (b) good, (c) poor Source: (A&B) [18,20,30,37,38], (C) [20,30,38]

Subjective evaluations were summarized in Table 5. No significant difference was seen in the number of patients reporting “excellent” (OR = 2.34, 95% CI: 0.75, 7.36; I^2^ = 79%) [18,20,30,37,38] or “poor” (OR = 0.72, 95% CI: 0.15, 3.41; I^2^ = 17%) [20,30,38]. However, there were more patients in the nonoperative group reporting “good” compared to the operative group (OR = 0.55, 95% CI: 0.33, 0.90; I^2^ = 80%) (Figure 6) [18,20,30,37,38].

**Table 5:** Reported dichotomous outcomes E: excellent; G: good; P: poor; SD: standard deviation; N: study sample; n: sample size; ACJ: acromioclavicular joint; OR: odds ratio

Outcomes	Events/sample size					
	Operative	Nonoperative	OR	95% CI	I^2^	Included study groups (N)
Return to work/previous activity	76/79	70/75	1.85	0.23-14.88	39%	3
Return to sports	67/83	62/73	0.73	0.30-1.81	0%	3
Subjective evaluation of results (E, G, P)	101/158, 36/158, 5/67	9/136, 48/136, 5/51	2.34, 0.55, 0.72	0.75-7.36, 0.33-0.90, 0.15-3.41	79%, 80%, 17%	5, 5, 3

Complications and Incidence of Surgery Posttreatment

Complications and incidence of surgery are summarized in Table 6. Nine papers reported complications in the operative group with 54 (18.3%) complications in total [17-20,32,33,36-38]. Common complications included infection (18.5%) [17-19,33,36-38] and osteosynthesis malfunction (16.7%) [17,32,33,38]. Eight studies reported adverse events in the nonoperative group with 39 (15.9%) adverse events [17-20,32,33,36,37]. A total of 26 (67%) referred to persistent symptoms [18,19,33,36]. Posttreatment, 28 (14%) patients in the nonoperative group converted to operative treatment [17-20,33,36], and 17 (7%) patients in the operative group required reoperation or revision surgery [17-19,32,33,36,38].

**Table 6:** Complications/adverse events and surgery posttreatment

Complications	Operative group		Nonoperative group	
	Events/sample size	Included study groups (N)	Events/sample size	Included study groups (N)
Total complications	54/295	9	39/245	8
Infection	10/251	7	-	-
Osteosynthesis malfunction	9/104	4	-	-
Needed reoperation/revision	17/241	7	-	-
Persistent symptoms	-	-	26/142	4
Converted to surgery	-	-	28/199	6

Discussion

Management of Rockwood III-V high-grade ACJ injuries remains contentious within the literature, with current guidance hinged on surgical opinion and patient preference. This review aimed to investigate and compare the functional, radiological, subjective, and return to activity or sports outcomes in patients with high-grade AC injuries treated conservatively and surgically. Here, it is shown that operative treatment demonstrates improved function and pain relief compared to nonoperative treatment at 24 to 48 months follow-up as indicated by Constant scores. Radiologically, operative treatment demonstrated a narrower ACJ width, while prospectively, it demonstrated a concomitant lower incidence of ACJ dislocation/subluxation at final follow-up between 24 and 48 months. Despite this, operative and nonoperative treatments were comparable for return to sports or activity. This meta-analysis, to the author’s knowledge, is the first to suggest that operative management of Rockwood III-V AC injuries appears to confer greater functional and radiological patient outcomes as opposed to nonoperative management. Surgical repair is thus a viable management option and should not preclude consideration in Rockwood III-V injury treatment where indicated.

The aim of treating AC injuries is to restore the normal anatomical alignment of the disrupted ACJ via clavicle reduction and immobilization. While conservative treatment is universally accepted for low-grade ACJ injuries [7], high-grade AC injuries remain controversial [22,40]. The primary finding of our study reports the Constant score being higher in the operative group at 24-48 months than in the conservative group. The Constant score (devised in 1987) measures several individual parameters including pain, activities of daily living, strength, and range of motion, together generating a global assessment of shoulder function to indicate the higher the score the higher the quality of function. A meta-analysis done on high-grade AC dislocations found the Constant score, which was reported in five studies, to be higher in the operative group than in the conservative group as well [16]. However, the follow-up period was not specified, and several classifications for AC separation were used. Three of our included studies reported a significant difference in the constant score in favor of the operative group at 24-48 months follow-up [32,35,38]. On the other hand, recent meta-analyses investigating Rockwood type III AC dislocations found no difference in constant scores between the two groups [41,42]. With our meta-analysis investigating specifically Rockwood grade III and above, our findings are the first to suggest that surgery provides better clinical outcomes at 24-48 months postoperation than conservative measures due to our included studies being the most up-to-date studies.

Conservative management is being reported as the initial management for grade III injuries which then may be converted to surgery especially in heavy laborers and elite athletes [4, 6]. A recent meta-analysis showed no significant difference in clinical outcomes in treating high-grade AC injuries surgically versus conservatively [16]. In addition, a study reported successful conservative treatment of active-duty service members with grade V injuries [43]. However, nonsurgical treatment does not address the anatomical displacement of the ACJ resulting in chronic ACJ dislocation. As such, disadvantages of nonsurgical treatment include shoulder instability and pain, cosmesis issues, and uncertainty of surgery in the long term which could be more challenging to perform due to the lack of ACJ reduction [44-46].

Operative management is usually encouraged due to its ability to restore joint anatomy and minimize long-term discomfort [47-49]. Rockwood III, IV, and V AC separations all involve the rupture of the AC and CC ligaments as well as the joint capsule with an associated detachment of deltoid and trapezius muscles seen only in IV and V injuries [5]. A more detailed breakdown of the Rockwood classification can be found in Supplementary Material 4. Both muscles are considered to be important stabilizers of the ACJ [50]. A randomized controlled trial [6] investigating the treatment of 12 type V ACJ injuries showed improved results with CC screw and ACJ fixation than conservative management. Furthermore, a recent case series on patients undergoing arthroscopic TightRope fixation for Rockwood III and V AC dislocations showed adequate CC ligament healing based on MRI evaluation and favorable functional outcomes [51]. In addition, upon our investigation of the incidence of adverse events after treatment, out of the 39 adverse events reported in the nonoperative group, 26 of them were due to consistent pain, unacceptable shoulder function, and cosmetic issues. Persistent symptoms and other causes can motivate patients to request conversion to a surgical approach, as reported by six of the studies included in this study [17-20,33,36]. To restore proper joint function and improve patient outcomes, surgery appears to be the superior approach. Further high-quality studies are needed to investigate long-term outcomes in patients comparing both surgical and nonsurgical management.

Our second finding is the difference in ACJ width at 48 months follow-up in favor of the operative group. This was supported by a prior meta-analysis that investigated Rockwood injuries of grade III only [41]. The normal ACJ width is known to be 1-6 mm in females and 1-7 mm in males [1]. Here, patients in the surgical group had a mean ACJ width of 5.41 mm while patients in the conservative group had a mean ACJ of 10.67mm. In addition, a reported lower incidence of ACJ dislocation in the operative group on radiological imaging was found compared to the nonoperative group. Both findings suggest that surgery provided a better joint reduction than conservative immobilization and rehabilitation. Given that our study also reported better functional outcomes, this might indicate a correlation between greater joint reduction and joint function. However, additional high-quality clinical trials need to be performed and explore this further.

Regarding the ability to return to sports, work, or previous activities at 24-48 months follow-up, our study reported no difference between the two groups. While this outcome gives some insight into patient recovery and treatment success, it omits sufficient indication of shoulder function and pain relief. An individual can return to previous activities/sports despite a persistently painful or reduced range of motion [52]. Furthermore, the types of activities, work, and sports vary among individuals and thus impact their quality of life differently. For the patients who did not return to previous activities, the included studies did not elaborate on the cause which might or might not be attributed to the injury itself. However, despite this, these findings demonstrate the viability of either operative or nonoperative measures if the return to activity is the main concern. Since the minority of our studies reported on the number of participants returning to work/sports, further studies should investigate the rate of return to previous activities, considering both occupation and sport, while also exploring the factors contributing to individuals not resuming their prior level of activity.

Limitations

As with all meta-analyses, limitations are present within the current study. Firstly, the majority of included studies did not report the baseline values of the outcomes of interest which limited assessment of the clinical significance of each treatment modality. The authors of the included studies were contacted three times with no response. Secondly, not all studies had a similar distribution of Rockwood III, IV, and V injuries, further contributing to heterogeneity. Thirdly, a follow-up time of up to six months might not be enough time to show clinical efficacy. Fourthly, the Constant score is not an ACJ-specific shoulder score which might not represent all ACJ injuries. Fifthly, none of the studies reported monitoring rehabilitation compliance and adherence, whether postoperative or nonoperative management, which might affect the functional outcome of the patients. Sixthly, in terms of study design, seven of the 13 included studies lacked a randomized controlled design which might affect the quality of results. To address all the limitations above, high-quality and standardized randomized control trials with a minimum of one-year follow-up need to be conducted investigating long-term outcomes of the management of Rockwood III-V ACJ injuries, preferentially for each grade in isolation.

## Conclusions

This meta-analysis demonstrated that operative management provides better shoulder function and pain relief at 24-48 months follow-up than nonoperative treatment in high-grade Rockwood III-V AC injuries. Patients undergoing operative management achieve improved ACJ reduction as indicated by narrower ACJ width and a lower incidence of ACJ dislocation/subluxation on radiographic imaging. Operative management is thus a viable option and may confer improved patient outcomes over conservative measures for high-grade AC injuries. Future studies with reported baseline values, prolonged follow‐up periods, larger sample sizes, and a homogenous treatment protocol are required to further assess the clinical difference between operative versus non-operative management of Rockwood III-V ACJ injuries.

## Appendices

Supplementary material 1: search strategies

Searches

PubMed 984

((Acromioclavicular OR "Acromioclavicular Joint"[Mesh] OR acromial joint OR articulatio acromioclavicularis OR acj) AND (injury OR dislocation OR luxation OR subluxation)) OR shoulder dislocation OR shoulder disarticulation OR rockwood

AND

 (surgery OR operat* OR surgical) AND ("Conservative Treatment"[Mesh] OR conservative treatment OR conservative therapy OR non-operative treatment OR conservative management OR nonoperative treatment OR non-surgical treatment OR nonsurgical treatment)

((("Acromioclavicular"[All Fields] OR "Acromioclavicular Joint"[MeSH Terms] OR ("acromial"[All Fields] AND ("joint s"[All Fields] OR "joints"[MeSH Terms] OR "joints"[All Fields] OR "joint"[All Fields])) OR ("Acromioclavicular Joint"[MeSH Terms] OR ("Acromioclavicular"[All Fields] AND "joint"[All Fields]) OR "Acromioclavicular Joint"[All Fields] OR ("articulatio"[All Fields] AND "acromioclavicularis"[All Fields]) OR "articulatio acromioclavicularis"[All Fields]) OR "acj"[All Fields]) AND ("injurie"[All Fields] OR "injuried"[All Fields] OR "injuries"[MeSH Subheading] OR "injuries"[All Fields] OR "wounds and injuries"[MeSH Terms] OR ("wounds"[All Fields] AND "injuries"[All Fields]) OR "wounds and injuries"[All Fields] OR "injurious"[All Fields] OR "injury s"[All Fields] OR "injuryed"[All Fields] OR "injurys"[All Fields] OR "injury"[All Fields] OR ("dislocate"[All Fields] OR "dislocated"[All Fields] OR "dislocates"[All Fields] OR "dislocating"[All Fields] OR "dislocator"[All Fields] OR "dislocators"[All Fields] OR "joint dislocations"[MeSH Terms] OR ("joint"[All Fields] AND "dislocations"[All Fields]) OR "joint dislocations"[All Fields] OR "dislocation"[All Fields] OR "dislocations"[All Fields]) OR ("luxated"[All Fields] OR "luxating"[All Fields] OR "luxation"[All Fields] OR "luxations"[All Fields]) OR ("joint dislocations"[MeSH Terms] OR ("joint"[All Fields] AND "dislocations"[All Fields]) OR "joint dislocations"[All Fields] OR "subluxation"[All Fields] OR "subluxations"[All Fields] OR "sublux"[All Fields] OR "subluxable"[All Fields] OR "subluxate"[All Fields] OR "subluxated"[All Fields] OR "subluxates"[All Fields] OR "subluxating"[All Fields] OR "subluxed"[All Fields] OR "subluxes"[All Fields] OR "subluxing"[All Fields]))) OR ("shoulder dislocation"[MeSH Terms] OR ("shoulder"[All Fields] AND "dislocation"[All Fields]) OR "shoulder dislocation"[All Fields]) OR (("shoulder"[MeSH Terms] OR "shoulder"[All Fields] OR "shoulders"[All Fields] OR "shoulder s"[All Fields]) AND ("disarticulated"[All Fields] OR "disarticulating"[All Fields] OR "disarticulation"[MeSH Terms] OR "disarticulation"[All Fields] OR "disarticulations"[All Fields])) OR ("rockwood"[All Fields] OR "rockwood s"[All Fields])) AND (("surgery"[MeSH Subheading] OR "surgery"[All Fields] OR "surgical procedures, operative"[MeSH Terms] OR ("surgical"[All Fields] AND "procedures"[All Fields] AND "operative"[All Fields]) OR "operative surgical procedures"[All Fields] OR "general surgery"[MeSH Terms] OR ("general"[All Fields] AND "surgery"[All Fields]) OR "general surgery"[All Fields] OR "surgery s"[All Fields] OR "surgerys"[All Fields] OR "surgeries"[All Fields] OR "operat*"[All Fields] OR ("surgical

procedures, operative"[MeSH Terms] OR ("surgical"[All Fields] AND "procedures"[All Fields] AND "operative"[All Fields]) OR "operative

surgical procedures"[All Fields] OR "surgical"[All Fields] OR "surgically"[All Fields] OR "surgicals"[All Fields])) AND ("Conservative Treatment"[MeSH Terms] OR ("Conservative Treatment"[MeSH Terms] OR ("conservative"[All Fields] AND "treatment"[All Fields]) OR "Conservative Treatment"[All Fields]) OR ("Conservative Treatment"[MeSH Terms] OR ("conservative"[All Fields] AND "treatment"[All Fields]) OR "Conservative Treatment"[All Fields] OR ("conservative"[All Fields] AND "therapy"[All Fields]) OR "conservative therapy"[All Fields]) OR ("non-operative"[All Fields] AND ("therapeutics"[MeSH Terms] OR "therapeutics"[All Fields] OR "treatments"[All Fields] OR "therapy"[MeSH Subheading] OR "therapy"[All Fields] OR "treatment"[All Fields] OR "treatment s"[All Fields])) OR ("Conservative Treatment"[MeSH Terms] OR ("conservative"[All Fields] AND "treatment"[All Fields]) OR "Conservative Treatment"[All Fields] OR ("conservative"[All Fields] AND "management"[All Fields]) OR "conservative management"[All Fields]) OR (("nonop"[All Fields] OR "nonoperative"[All Fields] OR "nonoperatively"[All Fields]) AND ("therapeutics"[MeSH Terms] OR "therapeutics"[All Fields] OR "treatments"[All Fields] OR "therapy"[MeSH Subheading] OR "therapy"[All Fields] OR "treatment"[All Fields] OR "treatment s"[All Fields])) OR (("nonsurgical"[All Fields] OR "nonsurgically"[All Fields]) AND ("therapeutics"[MeSH Terms] OR "therapeutics"[All Fields] OR "treatments"[All Fields] OR "therapy"[MeSH Subheading] OR "therapy"[All Fields] OR "treatment"[All Fields] OR "treatment s"[All Fields])) OR ("non-surgical"[All Fields] AND ("therapeutics"[MeSH Terms] OR "therapeutics"[All Fields] OR "treatments"[All Fields] OR "therapy"[MeSH Subheading] OR "therapy"[All Fields] OR "treatment"[All Fields] OR "treatment s"[All Fields]))))

EMBASE 1301

Embase

Session Results

.......................................................

No.  Query Results                                          Results  Date      

#25. #16 AND #24                                              1,301  29 Jun 2023

#24. #20 AND #23                                            289,067  29 Jun 2023

#23. #21 OR #22                                             764,005  29 Jun 2023

#22. 'conservative management' OR 'conservative              49,569  29 Jun 2023

     therapy' OR 'nonoperative treatment' OR

     'nonsurgical treatment'

#21. 'conservative treatment'/exp OR 'conservative          742,520  29 Jun 2023

     treatment'

#20. #17 OR #18 OR #19                                    9,280,069  29 Jun 2023

#19. surgical                                             2,393,882  29 Jun 2023

#18. operat*                                              2,122,148  29 Jun 2023

#17. 'surgery'/exp OR surgery                             8,063,548  29 Jun 2023

#16. #11 OR #14 OR #15                                       17,540  29 Jun 2023

#15. rockwood                                                 3,229  29 Jun 2023

#14. #12 OR #13                                              11,591  29 Jun 2023

#13. 'shoulder disarticulation'                                 106  29 Jun 2023

#12. 'shoulder dislocation'/exp OR 'shoulder                 11,532  29 Jun 2023

     dislocation'

#11. #5 AND #10                                               3,835  29 Jun 2023

#10. #6 OR #7 OR #8 OR #9                                 3,205,084  29 Jun 2023

#9.  'subluxation'                                           16,726  29 Jun 2023

#8.  luxation                                                 8,375  29 Jun 2023

#7.  dislocation                                             94,596  29 Jun 2023

#6.  'injury'/exp OR injury                               3,187,233  29 Jun 2023

#5.  #1 OR #2 OR #3 OR #4                                     5,065  29 Jun 2023

#4.  acj                                                        373  29 Jun 2023

#3.  'acromial joint' OR 'articulatio                             7  29 Jun 2023

     acromioclavicularis'

#2.  acromioclavicular                                        4,963  29 Jun 2023

#1.  'acromioclavicular joint'/exp OR                         3,989  29 Jun 2023

     'acromioclavicular joint'

Cochrane 129

Search Name: 

Date Run:        29/06/2023 06:47:45

Comment:      

ID        Search Hits

#1        MeSH descriptor: [Acromioclavicular Joint] explode all trees 84

#2        ("acromioclavicular"):ti,ab,kw (Word variations have been searched) 240

#3        (acromial joint):ti,ab,kw (Word variations have been searched)         41

#4        (articulatio acromioclavicularis):ti,ab,kw (Word variations have been searched)       0

#5        (acj):ti,ab,kw (Word variations have been searched)   35

#6        {OR #1-#5}      287

#7        MeSH descriptor: [Wounds and Injuries] explode all trees    34529

#8        (injury):ti,ab,kw (Word variations have been searched)          73921

#9        MeSH descriptor: [Joint Dislocations] explode all trees         935

#10      (dislocation):ti,ab,kw (Word variations have been searched) 2925

#11      (luxation):ti,ab,kw (Word variations have been searched)      139

#12      (subluxation):ti,ab,kw (Word variations have been searched) 584

#13      {OR #7-#12}    89807

#14      #6 AND #13    179

#15      MeSH descriptor: [Shoulder Dislocation] explode all trees    212

#16      (shoulder dislocation):ti,ab,kw (Word variations have been searched)          733

#17      (shoulder disarticulation):ti,ab,kw (Word variations have been searched)     0

#18      #15 OR #16 OR #17    733

#19      (rockwood):ti,ab,kw (Word variations have been searched)   95

#20      #18 OR #19     798

#21      #14 OR #20     890

#22      MeSH descriptor: [General Surgery] explode all trees            501

#23      (surgery):ti,ab,kw (Word variations have been searched)       267341

#24      (operat*):ti,ab,kw (Word variations have been searched)       124649

#25      (surgical):ti,ab,kw (Word variations have been searched)       124793

#26      {OR #22-#25}  345067

#27      MeSH descriptor: [Conservative Treatment] explode all trees            648

#28      ("conservative treatment"):ti,ab,kw (Word variations have been searched)   6581

#29      (conservative therapy OR non-operative treatment OR conservative management OR nonoperative treatment OR non-surgical treatment OR nonsurgical treatment):ti,ab,kw (Word variations have been searched)  18038

#30      #27 OR #28 OR #29    19847

#31      #26 AND #30  11572

#32      #21 AND #31  129

CiNAHL 218

#

Query

Limiters/Expanders

Last Run Via

Results

S20

S12 AND S19

Expanders - Apply equivalent subjects Search modes - Boolean/Phrase

Interface - EBSCOhost Research Databases Search Screen - Advanced Search Database - CINAHL Complete

218

S19

S15 AND S18

Expanders - Apply equivalent subjects Search modes - Boolean/Phrase

Interface - EBSCOhost Research Databases Search Screen - Advanced Search Database - CINAHL Complete

15,422

S18

S16 OR S17

Expanders - Apply equivalent subjects Search modes - Boolean/Phrase

Interface - EBSCOhost Research Databases Search Screen - Advanced Search Database - CINAHL Complete

22,292

S17

conservative treatment OR conservative therapy OR non-operative treatment OR conservative management OR nonoperative treatment OR non-surgical treatment OR nonsurgical treatment

Expanders - Apply equivalent subjects Search modes - Boolean/Phrase

Interface - EBSCOhost Research Databases Search Screen - Advanced Search Database - CINAHL Complete

22,292

S16

(MM "Conservative Treatment")

Expanders - Apply equivalent subjects Search modes - Boolean/Phrase

Interface - EBSCOhost Research Databases Search Screen - Advanced Search Database - CINAHL Complete

255

S15

S13 OR S14

Expanders - Apply equivalent subjects Search modes - Boolean/Phrase

Interface - EBSCOhost Research Databases Search Screen - Advanced Search Database - CINAHL Complete

1,065,722

S14

surgery OR operat* OR surgical

Expanders - Apply equivalent subjects Search modes - Boolean/Phrase

Interface - EBSCOhost Research Databases Search Screen - Advanced Search Database - CINAHL Complete

887,393

S13

(MM "Surgery, Operative+")

Expanders - Apply equivalent subjects Search modes - Boolean/Phrase

Interface - EBSCOhost Research Databases Search Screen - Advanced Search Database - CINAHL Complete

536,075

S12

S8 OR S9 OR S10 OR S11

Expanders - Apply equivalent subjects Search modes - Boolean/Phrase

Interface - EBSCOhost Research Databases Search Screen - Advanced Search Database - CINAHL Complete

3,929

S11

rockwood

Expanders - Apply equivalent subjects Search modes - Boolean/Phrase

Interface - EBSCOhost Research Databases Search Screen - Advanced Search Database - CINAHL Complete

962

S10

shoulder dislocation OR shoulder disarticulation

Expanders - Apply equivalent subjects Search modes - Boolean/Phrase

Interface - EBSCOhost Research Databases Search Screen - Advanced Search Database - CINAHL Complete

2,316

S9

(MM "Shoulder Dislocation")

Expanders - Apply equivalent subjects Search modes - Boolean/Phrase

Interface - EBSCOhost Research Databases Search Screen - Advanced Search Database - CINAHL Complete

1,532

S8

S3 AND S7

Expanders - Apply equivalent subjects Search modes - Boolean/Phrase

Interface - EBSCOhost Research Databases Search Screen - Advanced Search Database - CINAHL Complete

960

S7

S4 OR S5 OR S6

Expanders - Apply equivalent subjects Search modes - Boolean/Phrase

Interface - EBSCOhost Research Databases Search Screen - Advanced Search Database - CINAHL Complete

378,020

S6

injury OR dislocation OR luxation OR subluxation

Expanders - Apply equivalent subjects Search modes - Boolean/Phrase

Interface - EBSCOhost Research Databases Search Screen - Advanced Search Database - CINAHL Complete

377,875

S5

(MM "Subluxation")

Expanders - Apply equivalent subjects Search modes - Boolean/Phrase

Interface - EBSCOhost Research Databases Search Screen - Advanced Search Database - CINAHL Complete

1,114

S4

(MM "Dislocations+")

Expanders - Apply equivalent subjects Search modes - Boolean/Phrase

Interface - EBSCOhost Research Databases Search Screen - Advanced Search Database - CINAHL Complete

7,292

S3

S1 OR S2

Expanders - Apply equivalent subjects Search modes - Boolean/Phrase

Interface - EBSCOhost Research Databases Search Screen - Advanced Search Database - CINAHL Complete

1,424

S2

Acromioclavicular OR acromial joint OR articulatio acromioclavicularis OR acj

Expanders - Apply equivalent subjects Search modes - Boolean/Phrase

Interface - EBSCOhost Research Databases Search Screen - Advanced Search Database - CINAHL Complete

1,424

S1

(MM "Acromioclavicular Joint")

Expanders - Apply equivalent subjects Search modes - Boolean/Phrase

Interface - EBSCOhost Research Databases Search Screen - Basic Search Database - CINAHL Complete

665

Web of Science

# Searches:

1: TS=(Acromioclavicular OR acromial joint OR articulatio acromioclavicularis OR acj)                                                                    Results: 2992

2: TS=(injury OR dislocation OR luxation OR subluxation)                                                                 Results: 1352724

3: #2 AND #1                                                              Results: 1767

4: TS=(shoulder dislocation OR shoulder disarticulation)                                                                  Results: 7854

5: TS=(rockwood)                                                                    Results: 802

6: #3 OR #4 OR #5                                                                  Results: 9247

7: TS=(surgery OR operat* OR surgical)                                                                     Results: 5454901

8: TS=(conservative treatment OR conservative therapy OR non-operative treatment OR conservative management OR nonoperative treatment OR non-surgical treatment OR nonsurgical treatment)                                                                     Results: 121696

9: #7 AND #8                                                              Results: 76002

10: #6 AND #9                                                            Results: 838

Scopus 974

( ( TITLE-ABS-KEY ( surgery OR operat* OR surgical ) ) AND ( TITLE-ABS-KEY ( "conservative treatment" OR "conservative therapy" OR "non-operative treatment" OR "conservative management" OR "nonoperative treatment" OR "non-surgical treatment" OR "nonsurgical treatment" ) ) ) AND ( ( ( TITLE-ABS-KEY ( acromioclavicular OR "acromial joint" OR "articulatio acromioclavicularis" OR acj ) ) AND ( TITLE-ABS-KEY ( injury OR dislocation OR luxation OR subluxation ) ) ) OR ( TITLE-ABS-KEY ( "shoulder dislocation" OR "shoulder disarticulation" ) ) OR ( TITLE-ABS-KEY ( rockwood ) ) )

 Searches Update

((Acromioclavicular OR "Acromioclavicular Joint"[Mesh] OR acromial joint OR articulatio acromioclavicularis OR acj) AND (injury OR dislocation OR luxation OR subluxation)) OR shoulder dislocation OR shoulder disarticulation OR rockwood

AND

 (surgery OR operat* OR surgical) AND ("Conservative Treatment"[Mesh] OR conservative treatment OR conservative therapy OR non-operative treatment OR conservative management OR nonoperative treatment OR non-surgical treatment OR nonsurgical treatment)

26/6/23-31/12/24 68

Embase

Session Results

.......................................................

No.  Query Results                                          Results  Date      

#26. #16 AND #24 AND [29-6-2023]/sd NOT                         98  26 May 2024

     [1-1-2025]/sd

#25. #16 AND #24                                              1,378  26 May 2024

#24. #20 AND #23                                            306,376  26 May 2024

#23. #21 OR #22                                             804,866  26 May 2024

#22. 'conservative management' OR 'conservative              52,257  26 May 2024

     therapy' OR 'nonoperative treatment' OR

     'nonsurgical treatment'

#21. 'conservative treatment'/exp OR 'conservative          782,736  26 May 2024

     treatment'

#20. #17 OR #18 OR #19                                    9,773,810  26 May 2024

#19. surgical                                             2,524,975  26 May 2024

#18. operat*                                              2,254,706  26 May 2024

#17. 'surgery'/exp OR surgery                             8,479,784  26 May 2024

#16. #11 OR #14 OR #15                                       18,648  26 May 2024

#15. rockwood                                                 3,445  26 May 2024

#14. #12 OR #13                                              12,348  26 May 2024

#13. 'shoulder disarticulation'                                 109  26 May 2024

#12. 'shoulder dislocation'/exp OR 'shoulder                 12,289  26 May 2024

     dislocation'

#11. #5 AND #10                                               4,059  26 May 2024

#10. #6 OR #7 OR #8 OR #9                                 3,366,959  26 May 2024

#9.  'subluxation'                                           17,511  26 May 2024

#8.  luxation                                                 8,716  26 May 2024

#7.  dislocation                                             99,019  26 May 2024

#6.  'injury'/exp OR injury                               3,348,340  26 May 2024

#5.  #1 OR #2 OR #3 OR #4                                     5,367  26 May 2024

#4.  acj                                                        429  26 May 2024

#3.  'acromial joint' OR 'articulatio                             7  26 May 2024

     acromioclavicularis'

#2.  acromioclavicular                                        5,258  26 May 2024

#1.  'acromioclavicular joint'/exp OR                         4,243  26 May 2024

     'acromioclavicular joint'

.......................................................

Cochrane Library

Date Run:         27/05/2024 02:30:27

Comment:       

ID         Search  Hits

#1        MeSH descriptor: [Acromioclavicular Joint] explode all trees       89

#2        (acromioclavicular):ti,ab,kw (Word variations have been searched)          264

#3        (acromial joint):ti,ab,kw (Word variations have been searched)   49

#4        (articulatio acromioclavicularis):ti,ab,kw (Word variations have been searched)    0

#5        (acj):ti,ab,kw (Word variations have been searched)        40

#6        {OR #1-#5}        321

#7        MeSH descriptor: [Wounds and Injuries] explode all trees           38676

#8        (injury):ti,ab,kw (Word variations have been searched)   80513

#9        MeSH descriptor: [Joint Dislocations] explode all trees    1083

#10      (dislocation):ti,ab,kw (Word variations have been searched)        3150

#11      (luxation):ti,ab,kw (Word variations have been searched)            141

#12      (subluxation):ti,ab,kw (Word variations have been searched)       625

#13      {OR #7-#12}      98044

#14      #6 AND #13      197

#15      MeSH descriptor: [Shoulder Dislocation] explode all trees           231

#16      (shoulder dislocation):ti,ab,kw (Word variations have been searched)      794

#17      (shoulder disarticulation):ti,ab,kw (Word variations have been searched) 0

#18      #15 OR #16 OR #17       794

#19      ("Rockwood"):ti,ab,kw (Word variations have been searched)     114

#20      #18 OR #19       874

#21      #14 OR #20       973

#22      MeSH descriptor: [General Surgery] explode all trees      506

#23      (surgery):ti,ab,kw (Word variations have been searched) 292015

#24      (operat*):ti,ab,kw (Word variations have been searched) 135315

#25      (surgical):ti,ab,kw (Word variations have been searched) 136587

#26      {OR #22-#25}    375569

#27      MeSH descriptor: [Conservative Treatment] explode all trees      352

#28      ("conservative treatment"):ti,ab,kw (Word variations have been searched)          7149

#29      (conservative therapy OR non-operative treatment OR conservative management OR nonoperative treatment OR non-surgical treatment OR nonsurgical treatment):ti,ab,kw (Word variations have been searched)    19619

#30      #27 OR #28 OR #29       21558

#31      #26 AND #30    12596

#32      #21 AND #31    142

2023-2024 15

CiNAHL 220 limited to 2023-2024 14

#

Query

Limiters/Expanders

Last Run Via

Results

S20

S12 AND S19

Expanders - Apply equivalent subjects Search modes - Boolean/Phrase

Interface - EBSCOhost Research Databases Search Screen - Basic Search Database - CINAHL Complete

220

S19

S15 AND S18

Expanders - Apply equivalent subjects Search modes - Boolean/Phrase

Interface - EBSCOhost Research Databases Search Screen - Basic Search Database - CINAHL Complete

15,629

S18

S16 OR S17

Expanders - Apply equivalent subjects Search modes - Boolean/Phrase

Interface - EBSCOhost Research Databases Search Screen - Basic Search Database - CINAHL Complete

22,724

S17

conservative treatment OR conservative therapy OR non-operative treatment OR conservative management OR nonoperative treatment OR non-surgical treatment OR nonsurgical treatment

Expanders - Apply equivalent subjects Search modes - Boolean/Phrase

Interface - EBSCOhost Research Databases Search Screen - Basic Search Database - CINAHL Complete

22,724

S16

(MM "Conservative Treatment")

Expanders - Apply equivalent subjects Search modes - Boolean/Phrase

Interface - EBSCOhost Research Databases Search Screen - Basic Search Database - CINAHL Complete

463

S15

S13 OR S14

Expanders - Apply equivalent subjects Search modes - Boolean/Phrase

Interface - EBSCOhost Research Databases Search Screen - Basic Search Database - CINAHL Complete

1,080,679

S14

surgery OR operat* OR surgical

Expanders - Apply equivalent subjects Search modes - Boolean/Phrase

Interface - EBSCOhost Research Databases Search Screen - Basic Search Database - CINAHL Complete

897,565

S13

(MM "Surgery, Operative+")

Expanders - Apply equivalent subjects Search modes - Boolean/Phrase

Interface - EBSCOhost Research Databases Search Screen - Basic Search Database - CINAHL Complete

546,159

S12

S8 OR S9 OR S10 OR S11

Expanders - Apply equivalent subjects Search modes - Boolean/Phrase

Interface - EBSCOhost Research Databases Search Screen - Basic Search Database - CINAHL Complete

3,347

S11

rockwood

Expanders - Apply equivalent subjects Search modes - Boolean/Phrase

Interface - EBSCOhost Research Databases Search Screen - Basic Search Database - CINAHL Complete

383

S10

shoulder dislocation OR shoulder disarticulation

Expanders - Apply equivalent subjects Search modes - Boolean/Phrase

Interface - EBSCOhost Research Databases Search Screen - Basic Search Database - CINAHL Complete

2,300

S9

(MM "Shoulder Dislocation")

Expanders - Apply equivalent subjects Search modes - Boolean/Phrase

Interface - EBSCOhost Research Databases Search Screen - Basic Search Database - CINAHL Complete

1,519

S8

S3 AND S7

Expanders - Apply equivalent subjects Search modes - Boolean/Phrase

Interface - EBSCOhost Research Databases Search Screen - Basic Search Database - CINAHL Complete

977

S7

S4 OR S5 OR S6

Expanders - Apply equivalent subjects Search modes - Boolean/Phrase

Interface - EBSCOhost Research Databases Search Screen - Basic Search Database - CINAHL Complete

384,582

S6

injury OR dislocation OR luxation OR subluxation

Expanders - Apply equivalent subjects Search modes - Boolean/Phrase

Interface - EBSCOhost Research Databases Search Screen - Basic Search Database - CINAHL Complete

384,405

S5

(MM "Subluxation")

Expanders - Apply equivalent subjects Search modes - Boolean/Phrase

Interface - EBSCOhost Research Databases Search Screen - Basic Search Database - CINAHL Complete

1,114

S4

(MM "Dislocations+")

Expanders - Apply equivalent subjects Search modes - Boolean/Phrase

Interface - EBSCOhost Research Databases Search Screen - Basic Search Database - CINAHL Complete

7,378

S3

S1 OR S2

Expanders - Apply equivalent subjects Search modes - Boolean/Phrase

Interface - EBSCOhost Research Databases Search Screen - Basic Search Database - CINAHL Complete

1,407

S2

Acromioclavicular OR acromial joint OR articulatio acromioclavicularis OR acj

Expanders - Apply equivalent subjects Search modes - Boolean/Phrase

Interface - EBSCOhost Research Databases Search Screen - Basic Search Database - CINAHL Complete

1,407

S1

(MM "Acromioclavicular Joint")

Expanders - Apply equivalent subjects Search modes - Boolean/Phrase

Interface - EBSCOhost Research Databases Search Screen - Basic Search Database - CINAHL Complete

686

# Web of Science Search Strategy (v0.1)

# Database: Web of Science Core Collection

# Entitlements:

- WOS.IC: 1993 to 2024

- WOS.CCR: 1985 to 2024

- WOS.SCI: 1900 to 2024

- WOS.AHCI: 1975 to 2024

- WOS.BHCI: 2005 to 2024

- WOS.BSCI: 2005 to 2024

- WOS.ESCI: 2005 to 2024

- WOS.ISTP: 1990 to 2024

- WOS.SSCI: 1900 to 2024

- WOS.ISSHP: 1990 to 2024

# Searches:

1: TS=(Acromioclavicular OR acromial joint OR articulatio acromioclavicularis OR acj)                                            Date Run: Tue May 28 2024 13:02:33 GMT+1000 (Australian Eastern Standard Time)                   Results: 3211

2: TS=(injury OR dislocation OR luxation OR subluxation)                                         Date Run: Tue May 28 2024 13:03:04 GMT+1000 (Australian Eastern Standard Time)                       Results: 1459102

3: #1 AND #2                                      Date Run: Tue May 28 2024 13:03:32 GMT+1000 (Australian Eastern Standard Time)                     Results: 1890

4: TS=(shoulder dislocation OR shoulder disarticulation)                                          Date Run: Tue May 28 2024 13:03:57 GMT+1000 (Australian Eastern Standard Time)                       Results: 8444

5: TS=(rockwood)                                            Date Run: Tue May 28 2024 13:04:28 GMT+1000 (Australian Eastern Standard Time)                     Results: 872

6: #3 OR #4 OR #5                                          Date Run: Tue May 28 2024 13:16:11 GMT+1000 (Australian Eastern Standard Time)                     Results: 9941

7: TS=(surgery OR operat* OR surgical)                                             Date Run: Tue May 28 2024 13:16:33 GMT+1000 (Australian Eastern Standard Time)               Results: 5895137

8: TS=(conservative treatment OR conservative therapy OR non-operative treatment OR conservative management OR nonoperative treatment OR non-surgical treatment OR nonsurgical treatment)                                             Date Run: Tue May 28 2024 13:17:14 GMT+1000 (Australian Eastern Standard Time)                       Results: 132383

9: #7 AND #8                                      Date Run: Tue May 28 2024 13:17:46 GMT+1000 (Australian Eastern Standard Time)                     Results: 82918

10: #6 AND #9                                    Date Run: Tue May 28 2024 13:18:01 GMT+1000 (Australian Eastern Standard Time)                     Results: 897

11: #6 AND #9 and 2023 or 2024 (Publication Years)                                    Date Run: Tue May 28 2024 13:18:47 GMT+1000 (Australian Eastern Standard Time)                        Results: 72

Scopus

( ( TITLE-ABS-KEY ( surgery OR operat* OR surgical ) ) AND ( TITLE-ABS-KEY ( "conservative treatment" OR "conservative therapy" OR "non-operative treatment" OR "conservative management" OR "nonoperative treatment" OR "non-surgical treatment" OR "nonsurgical treatment" ) ) ) AND ( ( ( TITLE-ABS-KEY ( acromioclavicular OR "acromial joint" OR "articulatio acromioclavicularis" OR acj ) ) AND ( TITLE-ABS-KEY ( injury OR dislocation OR luxation OR subluxation ) ) ) OR ( TITLE-ABS-KEY ( "shoulder dislocation" OR "shoulder disarticulation" ) ) OR ( TITLE-ABS-KEY ( rockwood ) ) )

2023-2024 75

Supplementary material 2: PRISMA checklist

**Table 7:** PRISMA checklist PRISMA: preferred reporting items for systematic reviews and meta-analyses Source: [23]

Section and topic	Item #	Checklist item	Location where item is reported
TITLE			
Title	1	Identify the report as a systematic review.	1
ABSTRACT			
Abstract	2	See the PRISMA 2020 for Abstracts checklist.	4
INTRODUCTION			
Rationale	3	Describe the rationale for the review in the context of existing knowledge.	6-7
Objectives	4	Provide an explicit statement of the objective(s) or question(s) the review addresses.	7
METHODS			
Eligibility criteria	5	Specify the inclusion and exclusion criteria for the review and how studies were grouped for the syntheses.	8-9
Information sources	6	Specify all databases, registers, websites, organisations, reference lists, and other sources searched or consulted to identify studies. Specify the date when each source was last searched or consulted.	8
Search strategy	7	Present the full search strategies for all databases, registers, and websites, including any filters and limits used.	8
Selection process	8	Specify the methods used to decide whether a study met the inclusion criteria of the review, including how many reviewers screened each record and each report retrieved, whether they worked independently, and if applicable, details of automation tools used in the process.	8
Data collection process	9	Specify the methods used to collect data from reports, including how many reviewers collected data from each report, whether they worked independently, any processes for obtaining or confirming data from study investigators, and if applicable, details of automation tools used in the process.	8
Data items	10a	List and define all outcomes for which data were sought. Specify whether all results that were compatible with each outcome domain in each study were sought (e.g. for all measures, time points, analyses), and if not, the methods used to decide which results to collect.	10
	10b	List and define all other variables for which data were sought (e.g. participant and intervention characteristics, funding sources). Describe any assumptions made about any missing or unclear information.	
Study risk-of-bias assessment	11	Specify the methods used to assess risk of bias in the included studies, including details of the tool(s) used, how many reviewers assessed each study and whether they worked independently, and if applicable, details of automation tools used in the process.	8-9
Effect measures	12	Specify for each outcome the effect measure(s) (e.g. risk ratio, mean difference) used in the synthesis or presentation of results.	11-14
Synthesis methods	13a	Describe the processes used to decide which studies were eligible for each synthesis (e.g. tabulating the study intervention characteristics and comparing against the planned groups for each synthesis (item #5)).	
	13b	Describe any methods required to prepare the data for presentation or synthesis, such as handling of missing summary statistics, or data conversions.	
	13c	Describe any methods used to tabulate or visually display results of individual studies and syntheses.	
	13d	Describe any methods used to synthesize results and provide a rationale for the choice(s). If meta-analysis was performed, describe the model(s), method(s) to identify the presence and extent of statistical heterogeneity, and software package(s) used.	9
	13e	Describe any methods used to explore possible causes of heterogeneity among study results (e.g. subgroup analysis, meta-regression).	
	13f	Describe any sensitivity analyses conducted to assess robustness of the synthesized results.	
Reporting bias assessment	14	Describe any methods used to assess risk of bias due to missing results in a synthesis (arising from reporting biases).	
Certainty assessment	15	Describe any methods used to assess certainty (or confidence) in the body of evidence for an outcome.	
RESULTS			
Study selection	16a	Describe the results of the search and selection process, from the number of records identified in the search to the number of studies included in the review, ideally using a flow diagram.	11
	16b	Cite studies that might appear to meet the inclusion criteria, but which were excluded, and explain why they were excluded.	
Study characteristics	17	Cite each included study and present its characteristics.	11
Risk of bias in studies	18	Present assessments of risk of bias for each included study.	11-12
Results of individual studies	19	For all outcomes, present, for each study: (a) summary statistics for each group (where appropriate) and (b) an effect estimate and its precision (e.g. confidence/credible interval), ideally using structured tables or plots.	
Results of syntheses	20a	For each synthesis, briefly summarise the characteristics and risk of bias among contributing studies.	
	20b	Present results of all statistical syntheses conducted. If meta-analysis was done, present for each the summary estimate and its precision (e.g. confidence/credible interval) and measures of statistical heterogeneity. If comparing groups, describe the direction of the effect.	11-14
	20c	Present results of all investigations of possible causes of heterogeneity among study results.	
	20d	Present results of all sensitivity analyses conducted to assess the robustness of the synthesized results.	
Reporting biases	21	Present assessments of risk of bias due to missing results (arising from reporting biases) for each synthesis assessed.	
Certainty of evidence	22	Present assessments of certainty (or confidence) in the body of evidence for each outcome assessed.	
DISCUSSION			
Discussion	23a	Provide a general interpretation of the results in the context of other evidence.	15-18
	23b	Discuss any limitations of the evidence included in the review.	19
	23c	Discuss any limitations of the review processes used.	19
	23d	Discuss implications of the results for practice, policy, and future research.	19-20
OTHER INFORMATION			
Registration and protocol	24a	Provide registration information for the review, including register name and registration number, or state that the review was not registered.	8
	24b	Indicate where the review protocol can be accessed, or state that a protocol was not prepared.	
	24c	Describe and explain any amendments to information provided at registration or in the protocol.	
Support	25	Describe sources of financial or nonfinancial support for the review, and the role of the funders or sponsors in the review.	2
Competing interests	26	Declare any competing interests of review authors.	2
Availability of data, code and other materials	27	Report which of the following are publicly available and where they can be found: template data collection forms; data extracted from included studies; data used for all analyses; analytic code; any other materials used in the review.	2

Supplementary material 3: quality assessment

**Table 8:** Study quality assessment using the Newcastle Ottawa Scale

Study	Selection				Comparability	Outcome			Quality score
	Representativeness of the exposed cohort	Selection of the nonexposed cohort	Ascertainment of exposure	Demonstration that outcome of interest was not present at start of study	Comparability of cohorts on the basis of design or analysis	Assessment of outcome	Was follow-up long enough for outcomes to occur	Adequacy of follow-up of cohorts	
Álvarez-Álvarez et al., 2023 [30]	B*	A*	A*	A*	A*	B*	A*	A*	(8) Good
BoströmWindhamre et al., 2022 [18]	A*	A*	A*	A*	A**	A*	A*	B*	(9) Good
Canadian Orthopaedic Trauma Society, 2015 [17]	A*	A*	A*	A*	A**	A*	A*	D	(8) Good
Collaborative Orthopaedic Research Network, 2023 [31]	A*	A*	A*	A*	A*	B*	A*	C	(7) Good
De Carli et al., 2015 [37]	C	A*	A*	A*	A**	B*	A*	C	(7) Good
Gstettner et al., 2008 [38]	B*	A*	A*	A*	B*	B*	A*	B*	(8) Good
Joukainen et al., 2014 [33]	A*	A*	A*	A*	A**	A*	A*	B*	(9) Good
Mah et al., 2017 [34]	A*	A*	A*	A*	A**	A*	A*	D	(8) Good
Malik et al., 2021 [35]	A*	A*	A*	A*	A**	A*	A*	A*	(9) Good
Murray et al., 2018 [19]	B*	A*	A*	A*	A*	A*	A*	A*	(8) Good
Natera Cisneros et al., 2015 [32]	A*	A*	A*	A*	A*	B*	A*	A*	(8) Good
Saade et al., 2023 [20]	B*	A*	A*	A*	A**	B*	A*	C	(8) Good
Tauber et al., 2023 [36]	A*	A*	A*	A*	A*	A*	A*	A*	(8) Good

Supplementary material 4: Rockwood classification 

**Table 9:** Rockwood classification AC: acromioclavicular; CC: coracoclavicular

Grade	AC ligament	CC ligament	Joint capsule	Deltoid & trapezius	Radiological findings
I	Mild sprain	Intact	Intact	Intact	Normal
II	Ruptured	Sprain	Ruptured	Minimally detached	Increase of <25% in CC distance in relation to uninjured side
III	Ruptured	Ruptured	Ruptured	Detached	Increase between 25% and 100% in CC distance in relation to uninjured side
IV	Ruptured	Ruptured	Ruptured	Detached	Clavicle displaced posterior into the trapezius
V	Ruptured	Ruptured	Ruptured	Detached	Increase between 100% and 300% in CC distance in relation to uninjured side
VI	Ruptured	Ruptured	Ruptured	Detached	Clavicle displaced inferioposteriorly to coracobrachialis and biceps tendon
